# Rottweilers under primary veterinary care in the UK: demography, mortality and disorders

**DOI:** 10.1186/s40575-017-0051-7

**Published:** 2017-11-22

**Authors:** Dan G. O’Neill, Wee Yin Seah, David B. Church, Dave C. Brodbelt

**Affiliations:** 1 Pathobiology and Population Sciences, The Royal Veterinary College, Hawkshead Lane, North Mymms, Hatfield, Herts AL9 7TA UK; 2 Department of Clinical Services, Royal Veterinary College, Hawkshead Lane, Hatfield, Herts AL9 7TA UK

**Keywords:** VetCompass, Electronic patient record, EPR, Breed, Dog, Epidemiology, Primary-care, Veterinary, Pedigree, Purebred

## Abstract

**Background:**

Rottweilers are reportedly predisposed to many disorders but accurate prevalence information relating to the general population are lacking. This study aimed to describe demography, mortality and commonly recorded diseases in Rottweilers under UK veterinary care. Clinical health records within the VetCompass Programme were explored for disorders recorded during 2013.

**Results:**

Rottweilers comprised 5321 (1.17%) of 455,557 dogs attending 304 clinics. Annual proportional birth rates dropped from 1.75% in 2006 to 1.07% in 2013. Median adult bodyweight overall was 44.9 kg (IQR 39.55–51.00, range 20.00–88.80). Median male adult bodyweight (48.5 kg, interquartile range [IQR] 43.0–54.0, range 20.0–88.8) was heavier than female (41.5 kg, IQR 37.0–46.4, range 21.1–73.5) (*P* < 0.001). Median longevity overall was 9.0 years (IQR 7.2–10.5, range 0.0–17.0). Median female longevity (9.5 years, IQR 7.8–11.0) was greater than male (8.7 years, IQR 6.8–10.1) (*P* = 0.002). The most common causes of death were neoplasia (33.0%), inability to stand (16.0%) and mass-associated disorder (7.1%).

At least one disorder was recorded for 60.31% of Rottweilers. The most prevalent specific disorders recorded were aggression (7.46%, 95% CI 6.40–8.64), overweight/obesity (7.06%, 95% CI: 6.02–8.21), otitis externa (6.14%, 95% CI: 5.18–7.23) and degenerative joint disease (4.69%, 95% CI: 3.84–5.66). Male Rottweilers had higher prevalence than females for aggression (9.36% versus 5.47%, *P* = 0.001) and pyotraumatic dermatitis (4.05% versus 1.76%, *P* = 0.001). Aggression was more prevalent in neutered than entire females (7.5% versus 3.1%, *P* = 0.003) but did not differ between neutered and entire males (9.6% versus 9.0%, *P* = 0.773). The most frequent disorder groups were musculoskeletal (12.01%, 95% CI: 10.69–13.45), dermatological (10.96%, 95% CI: 9.69–12.35), gastro-intestinal (195, 8.87%, 95% CI: 7.72–10.14), undesirable behaviour (7.96%, 95% CI: 6.87–9.18) and neoplasia (7.96%, 95% CI: 6.87–9.18).

**Conclusions:**

The current study assists prioritisation of health issues within Rottweilers. Rottweilers are relatively short-lived and neoplasia is a common cause of death. The most common disorders were aggression, overweight/obesity, otitis externa and degenerative joint disease. Males were significantly heavier, shorter-lived and predisposed to aggression than females. These results can alert prospective owners to potential health issues and optimise sex selection decision-making.

## Plain English Summary

Rottweilers are reported as predisposed to musculoskeletal conditions, heart disease, parvoviral diarrhoea, cancers and uterine disease. The Rottweiler is over-represented among human dog bite-related fatalities and dog attacks on children. However, negative media stories may unfairly prime the public to perceive the breed as excessively aggressive.

Veterinary practice computer systems record large volumes of information and represent a useful source of research data. This study aimed to use veterinary data to describe the breed characteristics, mortality and most commonly recorded diseases in Rottweilers and specifically evaluate differences between females and males.

The VetCompass Programme collects data from UK veterinary practices for research. This study included dogs from the VetCompass Programme during 2013. Data on age, sex, neuter status and bodyweight were extracted. The cause, date and manner of all deaths was recorded. Clinical notes were reviewed manually to identify all disorders that existed during 2013.

From 455,557 dogs attending 304 clinics, there were 5321 (1.17%) Rottweilers. Adult males (48.5 kg) were heavier than adult females (41.5 kg). The Rottweiler dropped from 1.75% of all puppies born in 2006 to 1.07% in 2013 within dogs attending VetCompass practices. Average lifespan overall was 9.0 years. However, females (9.5 years) lived longer than males (8.7 years). The most common grouped causes of death were cancer (33.0%), inability to stand (16.0%) and lump-associated disorder (7.1%).

The most common specific disorders were aggression (7.46% of dogs), overweight/obesity (7.06%), ear infection (6.14%), and arthritis (4.69%). Males were more likely than females to have aggression (9.36% versus 5.47%) and acute skin infection (4.05% versus 1.76%). The most common disorder groups were joint (12.01% of dogs), skin (10.96%), gastro-intestinal (8.87%), undesirable behaviour (7.96%) and cancer (7.96%).

The Rottweiler appeared a relatively short-lived breed and cancer was a common cause of death. The most common disorders were aggression, overweight/obesity, ear infection and arthritis. Compared with female Rottweilers, males were significantly heavier, shorter-lived and predisposed to aggression. Awareness of breed health issues and sex-related differences may assist prospective owners to decide on a male versus female puppy.

## Background

The Rottweiler is believed to have originated from mastiff-type herding dogs taken north by the Roman army as they campaigned across Europe. In a town in southwest Germany called Rottweil, these dogs were mixed with sheepdog bloodlines to create the Rottweiler that was used for protecting property and their owners, herding and driving cattle, as well as pulling carts in the 19th century [1]. The onset of the industrial revolution resulted in a sharp decline in breed numbers but the Rottweiler regained popularity as a police and armed forces dog in the 1900s and the Rottweiler was exhibited at Crufts in 1936 [2–4]. However, evidence suggests that the breed has been in recent decline in the UK where annual Kennel Club (KC) registration counts for Rottweilers have dropped from 4257 in 2007 (1.6% of all registrations) to 1494 in 2016 (0.7% of all registrations) although data on breed numbers in the wider general population are scant [5].

Rottweilers are reported as predisposed to a number of health disorders [6]. Predisposition has been reported to a variety of musculoskeletal conditions including cruciate ligament disease, hip and elbow dysplasia, osteochondritis dissecans and osteosarcoma that may be associated with the rapid growth and large bodysize typical of the breed [7–9]. Other reported predispositions include dilated cardiomyopathy [10], parvovirus enteritis [11], lymphoma [12], histiocytic sarcoma [13] and cystic endometrial hyperplasia [14]. However, prevalence values for many of these disorders in the wider dog population are largely lacking [15]. This deficiency of reliable population-based prevalence data makes it problematic to apply an evidence based approach to scientifically prioritise health issues within the breed [16]. Despite this, breeders in the UK are currently strongly advised to participate in the BVA/KC Hip Dysplasia Scheme, BVA/KC Elbow Dysplasia Scheme and BVA/KC/ISDS Eye Scheme, and to test for Juvenile Laryngeal Paralysis and Polyneuropathy, before breeding Rottweliers [1].

Undesirable behavioral issues in Rottweilers have been the subject of considerable debate for many years and may be related to specific guarding characteristics deemed to be desirable in the breed [17, 18]. Studies in the US have reported that 16.3% of dog attacks on children involved Rottweilers [19] and 16.4% of human dog bite-related fatalities were ascribed to the Rottweiler [20]. The Rottweiler has been scored as high among breeds for aggression using behaviour-specific questionnaires [18, 21]. Male dogs have been reported as more likely to exhibit aggression than females [22]. Due to the strong natural guarding instincts of Rottweilers, the breed has become popular as a status dog with those seeking a macho image and has consequently suffered some bad publicity [1]. However, negative media stories may unfairly prime the public to perceive the breed as less approachable, more dangerous and aggressive than other breeds [23]. Unprompted information recorded on veterinary electronic patient records (EPRs) could represent another perspective to help elucidate a truer picture on undesirable behaviours in Rottweilers. Veterinary EPR data are increasingly being used to explore breed-based and disorder-based topics in dogs [24–26] and are reported as a useful data resource that is representative of the wider dog population [27].

The current study aimed to describe the demography, mortality and the most commonly recorded diseases in Rottweilers under veterinary care in the UK in order to extend the current evidence base supporting disorder prioritisation for improved health and welfare in the breed. Comparisons between females and males for demography and disorder prevalence were of particular interest in order to assist veterinarians and prospective owners to make evidence-based decisions on the sex selection within the breed.

## Methods

The study population included all dogs under primary veterinary care at clinics participating in the VetCompass Programme during 2013. Dogs under veterinary care were defined as those with either a) at least one EPR (VeNom diagnosis term, free-text clinical note, treatment or bodyweight) recorded during 2013 or b) at least one EPR recorded both before and after 2013. The VetCompass Programme collates de-identified EPR data from primary-care veterinary practices in the UK for epidemiological research [28]. Collaborating practices can record summary diagnosis terms during episodes of care from an embedded VeNom Code list [29]. Data fields available for VetCompass researchers include a unique animal identifier from each practice management system provider along with species, breed, date of birth, sex, neuter status, insurance status and bodyweight, and clinical information from free-form text clinical notes, summary diagnosis terms (VeNom codes) and treatment with relevant dates.

A prevalence study design derived from the cohort clinical data of dogs registered at participating practices was used to estimate the 1-year period prevalence of the most commonly diagnosed disorders [30]. Sample size calculations estimated that 2198 dogs would be needed to represent a disorder with 2.5% expected prevalence to a precision of 0.5% at a 95% confidence level from a population of 5321 dogs [31]. Ethics approval was obtained from the RVC Ethics and Welfare Committee (reference number 2016/U143).

Dogs recorded as Rottweiler breed were categorised as Rottweiler and all remaining dogs were categorised as non-Rottweiler. *All-age Bodyweight* (Kg) described recorded all available bodyweight and date combinations. *Adult Bodyweight* (Kg) described the maximum bodyweight recorded for dogs aged over 18 months and was categorised into six groups (< 30 kg, 30.0–39.9 kg, 40.0–49.9 kg, 50.0–59.9 kg, 60.0–69.9 kg, ≥ 70.0 kg). N*euter* described the status of the dog (entire or neutered) at the final EPR. *Age* described the age at the final date under veterinary care during 2013 and was defined at the earlier of December 31st, 2013 or the date of death.

The list of unique Rottweiler animal identification numbers was randomly ordered and a subset was reviewed manually in detail to extract the most definitive diagnostic term recorded for all disorders that existed during 2013 and to manually link this to the most appropriate VeNom term as previously described [32]. Elective (e.g. neutering) or prophylactic (e.g. vaccination) clinical events were not included. No distinction was made between pre-existing and incident disorder presentations. Disorders described within the clinical notes using presenting sign terms (e.g. ‘vomiting’ or ‘vomiting and diarrhoea’), but without a formal clinical diagnostic term being recorded, were included using the first sign listed (e.g. vomiting). Mortality data (recorded cause, date and method of death) were extracted on all deaths at any date during the available EPR data.

The extracted diagnosis terms were mapped to a dual hierarchy of precision for analysis: fine-level precision and grouped-level precision as previously described [32]. Briefly, fine-level precision terms described the original extracted terms at the maximal diagnostic precision recorded within the clinical notes (e.g. *inflammatory bowel disease* would remain as *inflammatory bowel disease*). Grouped-level precision terms mapped the original diagnosis terms to a general level of diagnostic precision (e.g. *inflammatory bowel disease* would map to *gastro-intestinal*).

Following data checking for internal validity and cleaning in Excel (Microsoft Office Excel 2013, Microsoft Corp.), analyses were conducted using Stata Version 13 (Stata Corporation). The sex, neuter status, age and adult bodyweight for Rottweilers under veterinary care during 2013 were described. Annual proportional birth rates described the relative proportion of Rottweilers compared with all dogs that were born in each year from 2006 to 2013 from the cohort that were under veterinary care in 2013. All-age bodyweight data with their associated dates were used to generate individual bodyweight growth curves for male and female Rottweilers by plotting age-specific bodyweights and were overlaid with a cross medians line plot using the Stata *mband* command.

One-year (2013) period prevalence values were reported along with 95% confidence intervals (CI) that described the probability of diagnosis at least once during 2013. The CI estimates were derived from standard errors based on approximation to the normal distribution for disorders with ten or more events [33] or the Wilson approximation method for disorders with fewer than ten events [34]. Prevalence values were reported overall and separately for males and females. The chi-square test to compare categorical variables and the Mann-Whitney U test to compare continuous variables [33]. Statistical significance was set at the 5% level.

## Results

### Demography and mortality

**Fig. 1:**
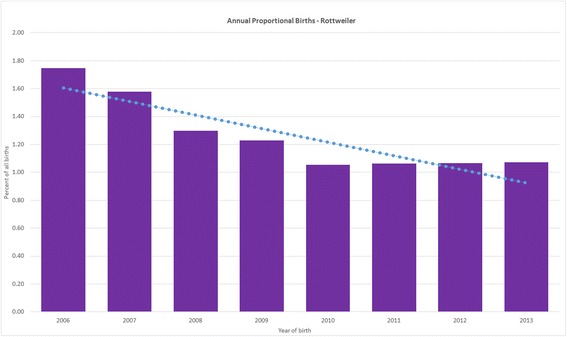
Annual proportional birth rates (2006–2013) for Rottweilers (*n* = 5321) among all dogs (*n* = 455,557) attending UK primary-care veterinary clinics participating in the VetCompass Programme

**Fig. 2:**
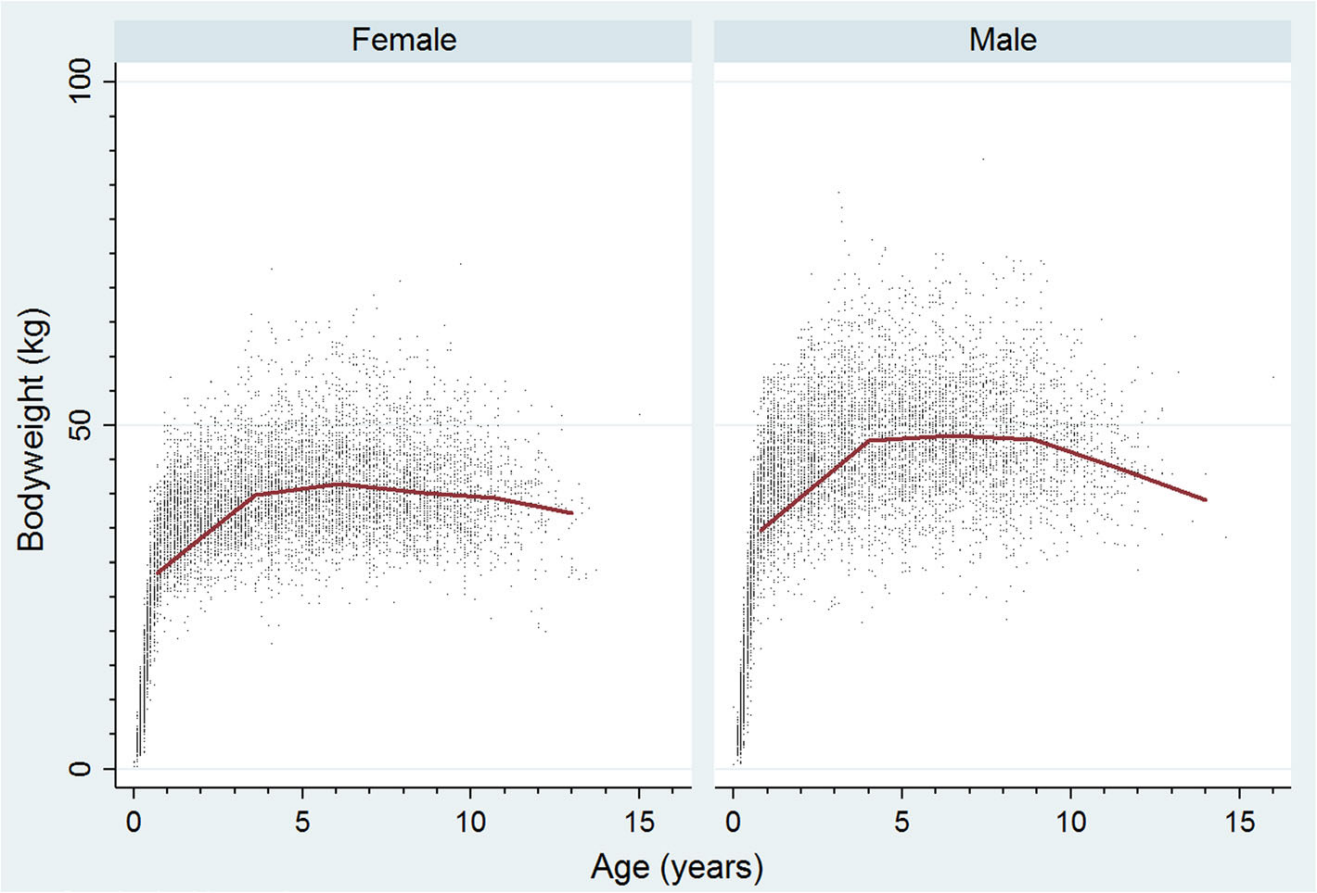
Bodyweight growth curves overlaid with a cross medians line prediction plot for female and male Rottweilers attending UK primary-care veterinary clinics participating in the VetCompass Programme. (Females *n* = 1898, Males *n* = 1829)

The study population of 455,557 dogs from 304 clinics in the VetCompass database under veterinary care during 2013 included 5321 (1.17%) Rottweilers. Of Rottweilers with information available, 2647 (49.9%) were female. Females were more likely to be neutered than males (45.8% versus 41.1%, *P* = 0.001). The median adult bodyweight overall was 44.9 kg (IQR 39.55–51.00, range 20.00–88.80). The median adult bodyweight of males (48.5, interquartile range [IQR] 43.0–54.0, range 20.0–88.8) was heavier than for females (41.5 kg, IQR 37.0–46.4, range 21.1–73.5) (*P* < 0.001). The median age of the Rottweilers overall was 4.5 years (IQR 1.9–7.5, range 0.0–17.0) (Table 1). Data completeness varied across the variables assessed: age 98.9%, sex 99.6%, neuter 85.0% and all-age bodyweight 85.9%. Annual proportional birth rates showed that Rottweilers dropped from 1.75% of the annual VetCompass birth cohort in 2006 to 1.07% in 2013 (Fig. 1). The median bodyweight across all ages for males (43.3 kg, IQR: 34.0–50.0, range: 0.6–88.8) was higher than for females (37.3 kg, IQR: 30.5–42.3, range: 0.3–73.5) (*P* < 0.001). Bodyweight growth curves based on 10,780 bodyweight values from 1897 females and 10,098 bodyweight values from 1829 males showed that Rottweiler puppies grow rapidly during their first year but that males plateau at a higher adult bodyweight than females (Fig. 2).

**Table 1:** Demography of Rottweilers under primary veterinary care at practices participating in the VetCompass Programme in the UK from January 1st, 2013 to December 31st, 2013 (*n* = 5321)

Variable	Category	No.	Percent
Sex	Female	2647	49.9
	Male	2653	50.1
Female neuter status	Entire	1234	54.2
	Neutered	1043	45.8
Male neuter status	Entire	1312	58.9
	Neutered	916	41.1
Female adult bodyweight (aged ≥18 months) (kg)	< 30.0	54	3.0
	30.0–39.9	664	36.3
	40.0–49.9	880	48.2
	50.0–59.9	198	10.8
	60.0–69.9	29	1.6
	≥ 70.0	2	0.1
Male adult bodyweight (aged ≥ 18 months) (kg)	< 30.0	16	0.9
	30.0–39.9	213	11.9
	40.0–49.9	752	42.0
	50.0–59.9	639	35.7
	60.0–69.9	150	8.4
	≥ 70.0	21	1.2
Age (years)	< 3.0	1876	35.6
	3.0–5.9	1370	26.0
	6.0–8.9	1307	24.8
	9.0–11.9	611	11.6
	≥ 12.0	101	1.9

There were 415 deaths recorded during the study. The median longevity of Rottweilers overall was 9.0 years (IQR 7.2–10.5, range 0.0–17.0). Of dogs with sex information available, the median longevity of females (9.5 years, IQR 7.8–11.0, range 0.3–14.2, *n* = 208) was greater than for males (8.7 years, IQR 6.8–10.1, range 0.0–17.0, *n* = 201) (*P* = 0.002). Overall, 91.3% of deaths with information available involved euthanasia and no significant difference in the method of death was identified between the sexes (*P* = 0.596). The median longevity of neutered animals (9.4 years, IQR 7.7–10.9, range 3.2–14.1) was longer than for entire animals (8.8 years, IQR 6.1–10.3, range 0.0–14.2) (*P* = 0.004). There were 103 (24.8%) deaths that did not have a cause of death stated. Of the remaining 312 deaths, the most common causes of death described at a grouped-precision level were neoplasia (*n* = 103, prevalence 33.0%), inability to stand (50, 16.0%) and mass-associated disorder (22, 7.1%). The probability of death did not differ between the sexes for any of the 10 most common causes of mortality (Table 2).

**Table 2:** Mortality in Rottweilers with a recorded cause of death under primary-care veterinary at UK practices participating in the VetCompass Programme from January 1st, 2013 to December 31st, 2013 (*n* = 312)

Grouped-level disorder	Overall Count (%)	Female count (%)	Male Count	*P*-Value male vs female
Neoplasia	103 (33.0)	52 (32.1)	51 (34)	0.690
Inability to stand	50 (16.0)	29 (17.9)	21 (14)	0.361
Mass-associated disorder	22 (7.1)	11 (6.8)	11 (7)	0.839
Gastro-intestinal	19 (6.1)	8 (4.9)	11 (7)	0.369
Brain disorder	13 (4.2)	8 (4.9)	5 (3)	0.486
Undesirable behaviour	13 (4.2)	5 (3.1)	8 (5)	0.315
Musculoskeletal disorder	12 (3.8)	8 (4.9)	4 (2)	0.303
Lower respiratory tract disorder	10 (3.2)	6 (3.7)	4 (2)	0.611
Spinal cord disorder	9 (2.9)	6 (3.7)	3 (2)	0.374
Vertebral arthropathy	8 (2.6)	4 (2.5)	3 (2)	0.787
Other	53 (12.8)			

The P-value reflects comparison between the prevalence in females and males

### Disorder prevalence

The EPRs of a random sample of 2197 (41.29%) of Rottweilers were manually examined to extract all recorded disorder data for 2013. There were 1325 (60.31%) Rottweilers with at least one disorder recorded during 2013 while the remaining 39.69% had no disorder recorded and either presented for prophylactic management only or did not present at all during 2013. The median count of disorders per Rottweiler during 2013 was one disorder (IQR 0–2, range 0–8). The median disorder count did not differ between females (median 1, IQR 0–2, range 0–8) and males (median 1, IQR 0–2, range 0–8) (*P* = 0.367).

The study included 2395 unique disorder events recorded during 2013 that encompassed 293 distinct fine-level disorder terms. The most prevalent fine-level precision disorders recorded were aggression (*n* = 164, prevalence 7.46%, 95% CI 6.40–8.64), overweight/obesity (155, 7.06%, 95% CI: 6.02–8.21), otitis externa (135, 6.14%, 95% CI: 5.18–7.23) and degenerative joint disease (103, 4.69%, 95% CI: 3.84–5.66). Among the 24 most common fine-level precision disorders, males were more likely than females to be diagnosed with aggression (9.36% versus 5.47% respectively, *P* = 0.001) and pyotraumatic dermatitis (4.05% versus 1.76% respectively, *P* = 0.001) (Table 3). Aggression was more likely in neutered than entire females (7.5% versus 3.1% respectively, *P* = 0.003) but aggression did not differ between neutered and entire males (9.6% versus 9.0% respectively, *P* = 0.773). Overweight/obesity was associated with neutering in both female (3.3% of entire versus 9.0% of neutered, *P* < 0.001) and male (4.7% of entire versus 11.8% of neutered, *P* < 0.001) Rottweilers.

**Table 3:** Prevalence of the most common disorders at a fine-level of diagnostic precision recorded in Rottweilers (*n* = 2197) attending UK primary-care veterinary practices participating in the VetCompass Programme from January 1st, 2013 to December 31st, 2013

Fine-level disorder	Count	Overall prevalence %	95% CI^a^	Female prevalence %	Male prevalence %	P-Value*
Aggression	164	7.46	6.40–8.64	5.47	9.36	0.001
Overweight/obesity	155	7.06	6.02–8.21	8.07	6.12	0.075
Otitis externa	135	6.14	5.18–7.23	6.31	6.03	0.787
Degenerative joint disease	103	4.69	3.84–5.66	5.19	4.23	0.287
Diarrhoea	77	3.50	2.78–4.36	3.25	3.78	0.498
Dental disease	67	3.05	2.37–3.86	3.06	3.06	0.999
Nail clip	67	3.05	2.37–3.86	3.15	2.97	0.803
Pyotraumatic dermatitis	64	2.91	2.25–3.70	1.76	4.05	0.001
Cruciate disease	51	2.32	1.73–3.04	2.69	1.98	0.271
Anal sac impaction	40	1.82	1.30–2.47	2.13	1.53	0.292
Conjunctivitis	40	1.82	1.30–2.47	1.48	2.16	0.238
Lameness	39	1.78	1.27–2.42	1.39	2.16	0.174
Vomiting	37	1.68	1.19–2.31	1.48	1.89	0.461
Lipoma	36	1.64	1.15–2.26	2.04	1.26	0.151
Skin mass	31	1.41	0.96–2.00	1.39	1.44	0.923
Urinary tract infection	30	1.37	0.92–1.94	1.86	0.90	0.055
Flea bite hypersensitivity	29	1.32	0.89–1.89	1.11	1.53	0.394
Wound	29	1.32	0.89–1.89	1.11	1.53	0.394
Pyoderma	28	1.27	0.85–1.84	1.3	1.26	0.936
Acrochordon	27	1.23	0.81–1.78	1.48	0.99	0.295
Hip dysplasia	27	1.23	0.81–1.78	1.11	1.35	0.616
Entropion	26	1.18	0.77–1.73	1.21	1.17	0.938
Haircoat disorder	25	1.14	0.74–1.68	1.02	1.26	0.598
Osteosarcoma - appendicular	25	1.14	0.74–1.68	1.02	1.26	0.598

*The P-value reflects prevalence comparison between females and males. ^a^CI confidence interval

There were 48 distinct grouped-level precision disorder terms recorded. The most prevalent grouped-level precision disorders were musculoskeletal (*n* = 264, prevalence: 12.01%, 95% CI: 10.69–13.45), dermatological (241, 10.96%, 95% CI: 9.69–12.35), gastro-intestinal (195, 8.87%, 95% CI: 7.72–10.14), undesirable behaviour (175, 7.96%, 95% CI: 6.87–9.18) and neoplasia (175, 7.96%, 95% CI: 6.87–9.18). Among the 15 most common grouped-level precision disorders, males were more likely than females to be diagnosed with dermatological (12.42% versus 9.46% respectively, *P* = 0.027) and undesirable behaviour disorders (9.81% versus 6.03% respectively, *P* = 0.001) while females were more likely than males to be diagnosed with urinary system disorders (3.62% versus 1.44% respectively, *P* = 0.001) (Table 4).

**Table 4:** Prevalence of the most common grouped-level disorders recorded in Rottweilers (*n* = 2197) attending UK primary-care veterinary practices participating in the VetCompass Programme from January 1st, 2013 to December 31st, 2013

Grouped-level disorder	Count	Overall prevalence	95% CI^a^	Female prevalence	Male prevalence	P-Value*I
Musculoskeletal	264	12.01	10.69–13.45	12.34	11.79	0.695
Dermatological	241	10.96	9.69–12.35	9.46	12.42	0.027
Gastro-intestinal	195	8.87	7.72–10.14	7.88	9.90	0.098
Undesirable behaviour	175	7.96	6.87–9.18	6.03	9.81	0.001
Neoplasia	175	7.96	6.87–9.18	8.26	7.74	0.657
Overweight/obesity	155	7.05	6.02–8.21	8.07	6.12	0.075
Aural	145	6.60	5.60–7.72	6.68	6.57	0.919
Claw/nail	109	4.96	4.09–5.95	5.10	4.86	0.795
Mass lesion	95	4.32	3.51–5.26	4.55	4.14	0.642
Dental	77	3.50	2.78–4.36	3.34	3.69	0.656
Traumatic injury	73	3.32	2.61–4.16	3.25	3.42	0.821
Ophthalmological	69	3.14	2.45–3.96	2.60	3.69	0.143
Urinary system	56	2.55	1.93–3.30	3.62	1.44	0.001
Anal sac	41	1.87	1.34–2.52	2.13	1.62	0.376
Congenital	41	1.87	1.34–2.52	1.76	1.98	0.707

*The P-value reflects prevalence comparison between females and males. ^a^CI confidence interval

## Discussion

This study offers the largest analysis of Rottweiler health reported to date and reports on breed-based disorders from 2197 Rottweilers under primary veterinary care in the UK. The most frequently recorded specific disorders were aggression, obesity and otitis externa, while the most common disorder groups were musculoskeletal, dermatological and gastro-intestinal. Male Rottweilers were more likely to have aggression and dermatological disorders than females. Accurate prevalence data on common disorders can provide a framework to facilitate disorder prioritisation in Rottweilers overall, while additional sex-based prevalence data can highlight those disorders that would benefit from special focus within specific sexes in order to contribute to improved Rottweiler health and welfare as well as assisting decision-making by veterinarians and owners on the most appropriate sex selection [15, 32].

The data used in the current study were collected from 304 primary-care veterinary clinics participating in the VetCompass Programme in the UK and aimed consequently to provide a representative view of the national UK health of the breed [28]. The application of veterinary clinical data for canine research has been proposed for many years as a unique opportunity for representative and generalisable health information relating to the wider dog population but technological issues have delayed the implementation of such research [15, 35]. In more recent times, the usefulness of veterinary EPR data for clinical research that can contribute to understanding of demography and clinical health in dogs has been demonstrated in several studies [7, 25, 26, 36]. Veterinary clinical data can benefit from reduced geographic selection bias by collecting from large numbers of clinics across the UK, from reduced patient selection bias by including all dog under veterinary care regardless of whether these have any health problems, from reduced misclassification and recall bias by using clinical information recorded contemporaneously by veterinarians during episodes of healthcare visits and from reduced disorder selection bias by including all disorders recorded in the clinical notes regardless of severity [27, 37, 38]. Establishment of consistent study methodologies also supports opportunities to repeat breed- and disease-specific studies in the future to provide reliable comparative data over time that can identify disorder occurrence trends and evaluate the effectiveness of any control measures put in place [26].

‘Big data’ collected from large counts of primary veterinary clinics now offers a unique resource to provide novel demographic perspectives on dogs [26]. Breed popularity trends for the Rottweiler appears to differ across the world. The proportion of Rottweilers registered with the UK KC has dropped from 1.6% of all registrations in 2007 to 0.7% in 2016 [5]. In contrast, the proportion of Rottweilers registered with the Australian KC has risen from 2.2% of all registrations in 2010 to 2.4% in 2016 [39] and the Rottweiler rose from the 9th most commonly registered breed in 2013 to the 8th rank in 2016 with the American KC [4]. Veterinary data offer a view on overall national breed statistics rather than focusing on just the pedigree registered subset. The results from the current study mirror the result from the UK KC and indicate that the Rottweiler has declined from 1.8% of all dogs born in 2006 to account for less than 1.2% in 2013. Reasons for rising and dropping popularity of individual breeds are complex and often appear counter-intuitive. Factors such as the distinctive appearance, health and behavioural problems, media publicity and longevity all play varying and often contrarian roles in the public perception [40, 41].

The mortality findings in this study highlighted neoplasia as the most common reason for death in Rottweilers, accounting for 33.0% of deaths. In addition, many of the 7.1% of deaths ascribed to mass-associated disorders may have been undiagnosed neoplasia, so the true impact of neoplastic-related deaths may be higher than reported here. By comparison, a study that also explored primary-care data in the UK, but over a longer timeframe, reported neoplasia as the cause of death in 16.5% of the overall population of dogs in England [42] These findings are higher than the results from a questionnaire study of pedigree dogs in Denmark that reported 20.4% mortality in Rottweilers from cancer [43] and a US study based on referral deaths which reported a cancer mortality of 28.2% [44]. A review of breeds predisposed to cancer identified that the Rottweiler is overrepresented for a number of neoplasia including osteosarcoma, histiocytic sarcoma and lymphoma [45]. Predisposition to osteosarcoma has especially been reported in several studies of the Rottweilers [46–48]. Such an osteosarcoma predisposition is supported by the inclusion of appendicular osteosarcoma within the list of common disorders with a prevalence of 1.14% reported by the current study. In consequence, veterinarians should consider osteosarcoma as a differential diagnosis for older Rottweilers presenting for lameness investigation, especially those with sudden and severe onset [49].

The median longevity of Rottweilers in the current study was 9.0 years which is shorter than the median longevity of 12.0 years reported across all breeds [42]. However, it is widely reported that average longevity reduces as breed bodysize increases [42, 50–55]. However, this 9.0 years longevity in Rottweilers compares poorly with the median longevity of 10.3 years reported for German Shepherd Dogs in the UK using a similar methodology and does suggest that the Rottweiler is a relatively short-lived breed [56].

The Rottweiler breed has been reported with predispositions to over 50 breed-related diseases [6]. Although extremely useful, these reports span a wide spectrum of countries and many of the reports are several decades old and therefore the generalisabilty of these reports to the current UK Rottweiler population is uncertain. In addition, predisposition to disease describes an over-representation of affected animals for that breed compared with the wider dog population but does not necessarily take into account other important factors such as absolute prevalence, duration and severity that determine the welfare impact of that condition [16]. To exemplify the differing perspectives generated by predisposition studies compared with prevalence studies, only five of the 52 disorders with previously reported predisposition in Rottweilers featured among the 24 most common disorders reported in the current study: pyotraumatic dermatitis, cruciate disease, hip dysplasia, entropion and appendicular osteosarcoma [6]. Many of the remaining disorders with reported predisposition that did not feature in the results of the current study may be quite rare and therefore their impact on breed welfare may be minimal at a population level. Conversely, although placement within the list of common disorders in the current study may imply a substantial welfare impact on the breed, it does not follow that Rottweilers are automatically predisposed for that disorder because certain disorders such as otitis externa and overweight/obesity are common across all dog breeds [32]. Reliable reporting on breed predispositions across a range of common disorders requires data that are directly comparable across breeds and disorders based on studies using a standard methodology. This paper is part of a series of breed studies that aims to provide comparative data to support robust predisposition analysis in the future [26, 56].

Canine aggression poses serious public health and animal welfare concerns [21]. Aggression was the most prevalent fine-level disorder identified in the current study, recorded in 7.46% of Rottweilers affected, while the broader group of undesirable behaviour overall was the fourth most common group of disorders with 7.96% of dogs affected. Based on a similar methodology, aggression did not feature among the 25 most common fine-level disorders in Pugs in the UK while undesirable behaviour was the 23rd most common group of disorders with a prevalence of 1.29% [26]. This wide variation between these two breeds is consistent with other reports of significant breed-related variation in aggression directed towards owners, strangers and other dogs that has been shown across a breadth of studies based on bite statistics, behavioural tests, referral caseloads and questionnaires [21, 22, 57–60]. The Rottweiler has specifically been reported with high propensity to aggression in a number of studies [17, 21, 61, 62] but other studies have not reported such predisposition for the Rottweiler [22]. However, it is worth noting that aggression includes a suite of behaviours that can be difficult to reliably and repeatedly characterise in dogs [21, 22]. The expression of various forms of aggression results from a complex interplay between genetic variation and the current and historical social environments experienced by individual dogs [21, 57, 61]. Paradoxically, fear may sometimes be associated with aggression although this relationship appears to vary across breeds; Rottweilers were more aggressive than fearful towards strangers whereas Greyhounds tended to show more fear than aggression [21]. Selection for working traits has been positively correlated with increased aggressiveness [57]. However, interpreting the true implications from studies on behaviour in general in dogs, and on aggression in particular, can be problematic. Owners of larger, powerful dogs are more likely to report problems and seek professional help in dealing with canine aggression because of the greater risk of injury posed by these dogs [63]. Behavioural ‘experts’ such as veterinarians, trainers and dog behaviourists may hold deeply ingrained beliefs about relative propensities for aggression across breeds and these stereotypes may colour their interpretations of certain behaviours [21]. Indeed, one study stated that ‘many dog professionals around the world consider the Rottweiler a very aggressive breed’ [62]. In summary however, although aggression is considered a complex disorder, the high prevalence recorded in the current study marks this behaviour out as a particular concern for the Rottweiler in the UK. Interestingly, results from selective breeding programmes against unwanted fear and aggression in the Netherlands offer the possibilities to reduce the prevalence of such tendencies by excluding dogs with unwanted fear and aggression from the Rottweiler breeding population [64].

This study aimed to explore differences in demography and disorder prevalence between females and males. Information on sex-related differences can assist veterinarians and prospective owners to better tailor selection decisions in order to choose a dog that best fits owners' needs. In consequence, these prior expectations and demands of the owner for their new dog’s behaviour and lifestyle may be associated with health outcomes during the later lives of these dogs [65]. The current study reports that adult male Rottweilers were on average 7 kg heavier than adult female Rottweilers. Whilst bodyweight conflates the effects of natural body conformation with any obesity issues, these results can assist prospective owners to match their expectations with the later reality of their dog’s size. Bodysize has multiple implications including financial cost of feeding and veterinary care, logistical issues around housing and travel by car, and also social acceptability [66]. The current study also reports that female Rottweilers live on average 9 months longer than male Rottweilers. Dogs are often important members of the human family system and grief and/or guilt following euthanasia or natural death of a cherished dog is well recognised [67–70]. Awareness of options to delay the probability of facing the decision and consequences of pet death by selecting a more long-lived sex may be a useful tool for owners and veterinarians.

The current study identified that males were significantly more likely to be diagnosed with aggression and pyotraumatic dermatitis than females. The finding of higher levels of aggression in males than female is supported by a substantial previous body of studies [58–61, 71–73]. Male dogs in general have also been reported to be more frequently involved in dog bite-related incidents compared with females [22, 74, 75]. Higher levels of aggression displayed by male dogs may result from the effects of androgens that promote dominant and competitive behaviour [71, 73]. Proportional neutering of Rottweilers does not appear to be differ substantially to the overall population of dogs of all types. The current study identified that neutering in 45.8% of female and 41.1%, male Rottweilers compared with 42.9% of female and 43.1% male dogs overall that were previously reported as neutered in England [32]. Although neutering is currently recommended in many countries to prevent undesirable behavioral problems [71, 76] the influence of sexual hormones in the arousal component of aggression is still controversial [22]. Associations between neutering and aggression reported from previous studies are inconsistent, varying from increased aggression [77] to no association [78, 79] to reduced aggression [60, 71, 80]. The effects of neutering on undesirable behaviours such as aggression are likely to be very complex and to vary across sex, breed, age at neutering, personality and environment [63, 81]. Although the current study reported a positive association between neutering and aggression in female but not in male Rottweiler, this does not imply causality as it is possible that aggressive individuals were more likely to be neutered in the first place. The results of the current study suggest that owners who desire a Rottweiler but who are concerned about possible aggression may be best to opt for a female rather than a male but do not support substantial benefits of reduced aggression from neutering.

The current study also showed a higher prevalence of pyotraumatic dermatitis in male (4.05%) compared with female (1.76%) Rottweilers. Pyotraumatic dermatitis was the eight most common fine-level disorder recorded and therefore contributes substantially to the disease burden for the breed. There is some previous evidence that Rottweilers, and males in particular, are over-represented for pyotraumatic dermatitis [82]. Also called acute moist dermatitis, ‘summer sores’ or ‘hot spot’, pyotraumatic dermatitis is an acute and rapidly developing surface bacterial skin infection secondary to self-inflicted trauma that adversely affects the welfare of affected animals which typically lick or scratch the affected area [83, 84]. Awareness of a sex predisposition in the breed may assist veterinarians and owners to target higher risk individuals for increased vigilance or prevention.

Overweight/obesity was the second most prevalent fine-level disorder overall in the current study, diagnosed in 7.06% of Rottweilers. It is possible that these results derived from secondary veterinary data may underestimate the true prevalence of overweight/obesity which has been reported as high as 25 to 44.4% in studies that were designed with an a priori focus on the disease [85–88]. A US study based on a primary-care veterinary caseload alone reported that just 1.4% of consultations in dogs had an *overweight* diagnosis code selected whereas the concurrent body condition score (BCS) indicated that 20.0% were overweight and suggested that practitioners may not perceive obesity/overweight as constituting a true disease state, especially for animals already in the overweight category [86]. Although the current study identifies overweight/obesity as a significant contributor to the overall disease burden, this prevalence value was just slightly higher than the values of 6.1 and 6.7% reported previously across all dog breeds in the UK in studies that were also reliant on primary-care veterinary clinical records [32, 89], therefore suggesting no particular predisposition to obesity in the Rottweiler. In contrast, US and French studies based on veterinary clinical data did identify some evidence for predisposition to obesity in the Rottweiler [86, 87]. Obesity is a highly complex trait and it may be that breed effects are heavily confounded with other risk factors, including age, sex, neutering, social, geographic and environmental influences that can markedly affect the occurrence of obesity [85, 86, 88, 90]. The results of the current study are consistent with earlier studies that identified substantial associations between neutering and obesity in both male and female dogs that should be considered carefully during the decision-making process on neutering for individual dogs [85, 86, 90]. Obesity control offers a significant opportunity for veterinary practices to improve the welfare of a large proportion of their canine caseload [90]. Successful weight management should embrace pro-active prevention strategies for the dogs of healthy weight was well as weight loss and maintenance protocols for those dogs already affected [91]. Prophylaxis or early intervention of obesity can potentially prevent diseases that are secondary to, or exacerbated by, this condition, including diabetes mellitus, cardiorespiratory, orthopedic, reproductive, dermatological disorders and anaesthetic complications [90].

Otitis externa was the most prevalent disorder (10.2%) recorded in dogs across all breeds in England [32] but was just the third most prevalent fine-level disorder at a prevalence of 6.14% in the current Rottweiler study. This differential ranking adds further emphasis to the relative importance of aggression and overweight/obesity in the Rottweiler but still identifies a substantial welfare burden from otic disorders in the Rottweiler. Although the current study recorded otitis externa as a single condition, the multiple facets to its underlying pathogenesis make it a very complex disorder with an aetiology involving many primary, perpetuating, predisposing and secondary factors [92]. Although breed predisposition has been reported in breeds such as the German Shepherd dog, Labrador retriever, Golden Retriever, West Highland White Terrier and Cocker Spaniel where atopic dermatitis was identified as a common primary cause of the otitis, the Rottweiler has not been highlighted as especially predisposed [93]. However, it may well be that there are individual primary, perpetuating, predisposing or secondary factors where the Rottweiler is specifically predisposed and therefore future exploration on the occurrence of otitis externa in the breed remains warranted.

Musculoskeletal disorders were ranked as the most common disorder group in the current study, affecting 12.01% of the Rottweiler study dogs. This high prevalence similar to the 11.8% value that was recorded across all breeds in England [32] and again highlights the paradox that absence of evidence for a breed predisposition should not be taken as evidence of absence for a substantial welfare impact. High welfare impact does not necessarily require predisposition but instead should be determined based upon prevalence, duration and severity metrics [16]. Among the musculoskeletal disorders group, the most common fine level disorder was degenerative joint disease which was recorded in 4.69% of Rottweilers. The main clinical presenting signs of degenerative joint disease are lameness, stiffness, exercise intolerance and/or an unwillingness/inability to climb or jump [94] which suggests that this condition impacts substantially on welfare and lifestyle. The UK KC currently recommends that scores from the BVA/KC Hip Dysplasia Scheme and BVA/KC Elbow Dysplasia Scheme contribute to breeding decisions for Rottweilers but it may worth also considering other functional joint testing in addition as part of this process [1].

This study had some limitations that have been reported previously [32, 56]. Typical of commonly accepted protocols in primary-care veterinary practice, many disorders in the current study were clinically managed without progressing to a final specified diagnosis due to possible limiting factors such as time or financial constraints, and limited laboratory testing, referral or post-mortem examination [25]. A primary-care observational study in the UK reported that just 31.8% of new health problems reached a definitive diagnosis [89]. In the current study, this effect was notable by separate reporting for neoplastic and mass-associated disorders in Tables 2 and 4, while in reality, many of the mass-associated disorders may truly be neoplastic in origin. Disorder information depended on the diagnostic acumen and note-taking of the clinicians involved in the study [32]. The present study reported prevalence of disorders but effective prioritisation of welfare would require additional data on severity and duration [16]. It is possible that some animals moved between clinics during the study period and may therefore have been duplicated in the denominator population.

## Conclusion

The current study extends the evidence base on the demography, mortality and disorders of Rottweilers and provides a valuable framework to assist with prioritisation of health issues within the breed. The breed is shown to be relatively short-lived and neoplasia is identified as a common cause of death. The most common disorders diagnosed were aggression, overweight/obesity, otitis externa and degenerative joint disease. Compared with female Rottweilers, males were significantly heavier, shorter-lived and predisposed to aggression. Awareness of the breed-based and sex-related differences may assist prospective owners during consideration of the Rottweiler as a breed-type to acquire per se as well as to optimise sex selection decision-making.

## Abbreviations

AKC: The American Kennel Club
CI: Confidence interval
EPR: Electronic patient record
IQR: Interquartile range
KC: The Kennel Club
OR: Odds ratio
